# Impact of Biologic Therapy on the Small Airways Asthma Phenotype

**DOI:** 10.1007/s00408-022-00579-2

**Published:** 2022-10-14

**Authors:** Rory Chan, Brian J. Lipworth

**Affiliations:** Scottish Centre for Respiratory Research, Ninewells Hospital and Medical School, University of Dundee, Dundee, Scotland DD1 9SY UK

**Keywords:** Small airways, Severe asthma, FEF_25–75_, Oscillometry, Multiple breath nitrogen washout, Omalizumab, Mepolizumab, Reslizumab, Benralizumab, Dupilumab, Tezepelumab, Itepekimab

## Abstract

The small airways dysfunction (SAD) asthma phenotype is characterised by narrowing of airways < 2 mm in diameter between generations 8 and 23 of the bronchial tree. Recently, this has become particularly relevant as measurements of small airways using airway oscillometry for example, are strong determinants of asthma control and exacerbations in moderate-to-severe asthma. The small airways can be assessed using spirometry as forced expiratory flow rate between 25 and 75% of forced vital capacity (FEF_25–75_) and has been deemed more accurate in detecting small airways dysfunction than forced expiratory volume in 1 s (FEV_1_). Oscillometry as the heterogeneity in resistance between 5 and 20 Hz (R5–R20), low frequency reactance at 5 Hz (X5) or area under the reactance curve between 5 Hz and the resonant frequency can also be used to assess the small airways. The small airways can also be assessed using the multiple breath nitrogen washout (MBNW) test giving rise to values including functional residual capacity, lung clearance index and ventilation distribution heterogeneity in the conducting (Scond) and the acinar (Sacin) airways. The ATLANTIS group showed that the prevalence of small airways disease in asthma defined on FEF_25–75_, oscillometry and MBNW all increased with progressive GINA asthma disease stages. As opposed to topical inhaler therapy that might not adequately penetrate the small airways, it is perhaps more intuitive that systemic anti-inflammatory therapy with biologics targeting downstream cytokines and upstream epithelial anti–alarmins may offer a promising solution to SAD. Here we therefore aim to appraise the available evidence for the effect of anti-IgE, anti-IL5 (Rα), anti-IL4Rα, anti-TSLP and anti-IL33 biologics on small airways disease in patients with severe asthma.

## Introduction

The small airways dysfunction (SAD) asthma phenotype is characterised by narrowing of airways < 2 mm in diameter between generations 8 and 23 of the bronchial tree [1]. Recently, this has become particularly relevant as measurements of small airways using airway oscillometry for example, are strong determinants of asthma control and exacerbations in moderate-to-severe asthma [2]. The small airways can be assessed using spirometry as forced expiratory flow rate between 25 and 75% of forced vital capacity (FEF_25–75_) and has been deemed more accurate in detecting small airways dysfunction than forced expiratory volume in 1 s (FEV_1_) [3]. Having said that FEF_25–75_ is rather volume dependent in terms of ensuring patients breathe out all the way to residual volume and as such is considered to be more variable.

Oscillometry as the heterogeneity in resistance between 5 and 20 Hz (R5–R20), low frequency reactance at 5 Hz (X5) or area under the reactance curve (AX) between 5 Hz and the resonant frequency can also be used to assess the small airways [4]. X5 and AX are thought to reflect peripheral lung compliance which is reduced in patients with SAD. Oscillometry has advantages over spirometry in being effort independent, being more associated with type 2 inflammation and having higher sensitivity with regards to bronchodilator responses [5, 6]. In patients with moderate-to-severe persistent asthma not taking biologics the presence of abnormal values of either R5–R20 ≥ 0.10 kPa/L/s or AX ≥ 1.0 kPa/L were associated with worse disease control as ACQ score [7]. Using computational modelling, it has previously been shown that R5–R20 is a direct measure of anatomical narrowing of the small airways [8]. Contemporaneously, it has been determined that combining both spirometry and oscillometry measurements might better identify moderate-to-severe asthma patients with worse control and more frequent exacerbations [9, 10].

The small airways can also be assessed using the multiple breath nitrogen washout (MBNW) test giving rise to values including functional residual capacity, lung clearance index and ventilation distribution heterogeneity in the conducting (Scond) and the acinar (Sacin) airways [11]. The ATLANTIS group showed that the prevalence of small airways disease in asthma defined on FEF_25–75_, oscillometry and MBNW all increased with progressive GINA asthma disease stages [12].

The peripheral airways have previously been termed the quiet zone of the lung because they are difficult to assess and treat. Conventional high doses of inhaled corticosteroids have been shown to be relatively ineffective in managing distal lung inflammation measured by alveolar nitric oxide [13]. This is likely attributed to aerosols comprising a larger particle size that have a predilection to deposit in the large airways [14]. In one study, adding extra-fine HFA–BDP on top of high dose conventional particle fluticasone/salmeterol conferred no improvement in oscillometry small airways function or alveolar NO in patients with severe persistent asthma [15]. Over the past decade, type 2 biologic therapies have been shown to significantly improve exacerbations and other clinical outcomes such as disease control, pulmonary function and type 2 biomarkers [16, 17].

Here we therefore aim to appraise the available evidence for the effect of systemic biologic therapies on small airways disease in patients with severe asthma. We searched PubMed and Google Scholar for terms including “small airways”, “omalizumab”, “mepolizumab”, “benralizumab”, “reslizumab”, “dupilumab”, “tezepelumab”, “itepekimab”, “FEF_25–75_”, “oscillometry” and “multiple breath nitrogen washout” with abstracts and case reports excluded. The aim here is not to perform a systematic review or meta–analysis as the investigated outcomes in these cited studies are too heterogenous to amalgamate. The essential premise here is that the systemic route of administration would facilitate delivery of biologics to the whole lung including the peripheral airways in the same way as oral corticosteroids in patients who are refractory to high dose ICS. Given that the airway mucosal surface area is proportionately much greater in the distal compared to proximal lung, systemic delivery of biologics appears to be a cogent way for treating all of the type 2 inflammation in asthmatic airways. Indeed, this may be one of the reasons why systemic biologics are so successful at improving control in severe asthma patients despite the use of high dose inhaled combination therapy.

### Omalizumab

Omalizumab is a recombinant humanised anti-IgE monoclonal IgG1 antibody that blocks the binding of free IgE to its high affinity FcεRI receptor on mast cells and basophils [18]. It has the secondary action of binding to membrane bound IgE (mIgE) on mIgE-expressing B cells resulting in downregulation of IgE production [19]. A Cochrane review has demonstrated significant reductions in exacerbations and hospitalisations in moderate-to-severe asthma [20]. As FcεRI expression is increased throughout the large and small airways in severe asthma [21], one might postulate that a systemic therapy such as omalizumab would confer additional benefit to allergic patients only taking topical inhaler therapy.

A retrospective cohort study (*n* = 110) in adult patients with severe eosinophilic allergic asthma showed that omalizumab significantly improved FEF_25–75_ by 8.3% over 52 weeks [22]. Another real-life retrospective clinical study (*n* = 20) of severe asthma patients demonstrated that omalizumab significantly improves FEF_25–75_% by 6% but not FEV_1_% by 4%, over 44 weeks along with clinically significant reductions in exacerbations and ACQ scores [23]. A prospective observational study (*n* = 26) also highlighted an improvement in alveolar nitric oxide levels in severe asthmatics following 48 weeks of omalizumab indicating a potential therapeutic effect on small airways type 2 inflammation [24]. This is important as uncontrolled small airways inflammation is related to airway remodelling and progression of disease [25]. Additionally, patients with aspirin exacerbated respiratory disease generally have higher levels of type 2 inflammation [26], and in one small case series (*n* = 4) such patients also experienced improvements in FEF_25–75_% by 30% [27]. No studies have been performed looking at the effect of omalizumab therapy on other measures of small airways disease.

### Mepolizumab, Reslizumab and Benralizumab

Mepolizumab and Reslizumab are humanised IgG1κ and IgG4κ monoclonal antibodies, respectively, that exert its effect by inhibiting interleukin 5 attachment to the IL5Rα receptor on eosinophils [28, 29]. Benralizumab is a humanised IgG1κ monoclonal antibody that binds to the IL5Rα receptor on eosinophils to prevent IL5 activation [30]. Through this shared mechanism of action, suppression or depletion of eosinophilic activation, proliferation and migration is achieved. Due to higher expression of IL5 mRNA in the small airways (< 2 mm diameter) in asthmatics, one might expect mepolizumab, reslizumab and benralizumab therapy to be effective in SAD [31].

The phase 3b RCT MUSCA demonstrated significant improvements in FEF_25–75_ amounting to 0.123 L/s after 24 weeks of mepolizumab vs placebo in *n* = 551 patients with severe eosinophilic asthma [32]. Although this is the largest study investigating the effect of mepolizumab in small airways, MUSCA was not powered a priori on FEF_25–75_ [32]. To support this, two retrospective studies (*n* = 134 and *n* = 105) independently demonstrated a significant improvement in FEF_25–75_% with mepolizumab in severe eosinophilic asthma patients with respective improvements of 9.8% and 8.1% [33, 34]. Smaller observational studies [23, 35] (*n* = 31 and *n* = 30) have shown no improvement in FEF_25–75_% after 24–44 weeks of mepolizumab. However, the mean baseline FEF_25–75_% in these smaller studies were higher and therefore there may have been less room for improvement.

In a prospective study (*n* = 18), it was shown that oscillometry low frequency reactance as X5, a measure of peripheral lung compliance, significantly improved by 74% one month post mepolizumab therapy in severe eosinophilic asthma [36]. However, another retrospective study in severe asthmatics (*n* = 30) showed no improvements in R5–R20 or AX following 10 months of mepolizumab [23]. These studies are likely to be underpowered to draw any meaningful conclusions. One prospective cohort study (*n* = 20) showed a significant improvement in small airway function after 26 weeks with mepolizumab measured by ventilation heterogeneity as Sacin using MBNW in patients with severe eosinophilic asthma [37].

In a phase 3 randomised controlled trial (RCT) (*n* = 205) [38], there was a borderline significant trend for iv reslizumab 3 mg/kg to improve FEF_25–75_ over 16 weeks by 0.233 L/s vs placebo, exceeding the established biological variability in severe asthma of 0.21 L/s [39] to infer a clinically relevant treatment effect. An open label extension study (*n* = 1051) has shown that these FEF_25–75_% improvements persist up to 96 weeks on reslizumab in patients with moderate-to-severe eosinophilic asthma [40]. Post hoc analysis of 2 phase 3 RCTs (*n* = 723) in severe eosinophilic asthma showed that reslizumab significantly improves FEF_25–75_ over placebo with a mean difference 0.128 L/s [41]. Although reslizumab is used in clinical practice to a lesser extent, we postulate that these encouraging results can possibly be extrapolated to mepolizumab due to the shared immunological pathway. No studies to date have been performed on reslizumab looking at oscillometry or MBNW outcomes.

A multicentre retrospective observational study [42] (*n* = 137) looking at patients with severe eosinophilic asthma demonstrated significant improvements in FEF_25–75_% amounting to 17% after 24 weeks of benralizumab. Another real-life retrospective observational study [43] (*n* = 22) showed that benralizumab improved FEF_25–75_ by 0.82 L/s over 24 weeks in severe allergic eosinophilic asthma patients greatly exceeding the biological variability value [39] for a clinically relevant effect. In one prospective observational study with benralizumab in severe asthma [44] (*n* = 19) no improvements in R5–R20, X5 and AX were observed after 24 weeks. Pointedly, patients in this study started with normal small airways function and therefore one would perhaps not expect any improvement.

### Dupilumab

Dupilumab is a humanised IgG4 monoclonal antibody that targets the IL4Rα receptor to mediate IL4 and IL13 activity [45]. Interestingly, IL4 and IL13 but not IL5 have been shown to induce hyperresponsiveness in isolated small airways [46]. Additionally, more IL4 mRNA expression has been found in the small airways of asthmatic versus non-asthma patients [31].

The phase 3 LIBERTY ASTHMA QUEST trial [47] (*n* = 1902) in uncontrolled moderate-to-severe asthma showed that FEF_25–75_ significantly improved by 0.16 L/s following 52 weeks of dupilumab treatment compared to placebo. In this regard, a phase 2 RCT [48] (*n* = 148) in moderate-to-severe asthma also showed that dupilumab improved FEF_25–75_ by 0.19 L/s compared to placebo over 12 weeks albeit the significance was not reported here since it was not the primary outcome. In another prospective cohort study [49] (*n* = 20) of severe asthma patients with nasal polyps treated with dupilumab for 4 weeks there was a significant improvement in FEF_25–75_ of 0.33 L/s exceeding biological variability. In terms of airway oscillometry, one retrospective study [50] (*n* = 62) in mild-to-moderate asthma with concomitant CRSwNP showed that 3 months of dupilumab therapy did not significantly change X5.

### Tezepelumab

Tezepelumab is a humanised IgG2*λ* monoclonal antibody that blocks the upstream epithelial alarmin thymic stromal lymphopoietin (TSLP) from interacting with the TSLP receptor complex resulting in dampening of the type 2 inflammatory response [51]. The phase 2 CASCADE trial [52] (*n* = 110) in moderate-to-severe uncontrolled asthma demonstrated no improvement in FEF_25–75_ or R5–R20 over placebo although interestingly tezepelumab resulted in a 0.56 kPa/L improvement in AX that exceeds the biological variability value of 0.39 kPa/L in severe asthma [39].

### Itepekimab

Itepekimab is a humanised IgG4 monoclonal antibody with anti-alarmin activity against IL-33 resulting in suppression of type 2 inflammation [48]. In a phase 2 RCT of moderate-to-severe asthmatics (*n* = 148) [48], itepekimab was shown to improve FEF_25–75_ by 0.170 L/s over 12 weeks compared to placebo, which did not exceed the biological variability value. In this regard, the same phase 2 RCT [48] with combined itepekimab and dupilumab conferred a 0.120 L/s improvement in FEF_25–75_ over placebo which was numerically less than for itepekimab or dupilumab monotherapy alone. This suggests that merely blocking more type 2 inflammatory pathways may not be the answer. The effect of various biologic therapies on FEF_25–75_ is summarised in tabular form (Table 1).

**Table 1:** Summary of current evidence base for the effect of biologics on forced expiratory flow rate between 25 and 75% of forced vital capacity (FEF_25–75_)

Study	Type of study	Biologic	Numbers of patients	Baseline FEF_25–75_ (L/s or %)	Duration of therapy (weeks)	Absolute improvement (L/s or %)^a^	*P* value
Huang et al. [22]	Retrospective	OMA	*n* = 110	55.1%	52	8.3%	< 0.001
Chan et al. [23]	Retrospective	OMA	*n* = 20	43%	44	6%	< 0.05
Chupp et al. [32]	RCT	MEPO	*n* = 551	0.894 L/s	24	0.123 L/s	0.002
Sposato et al. [33]	Retrospective	MEPO	*n* = 134	37.4%	47	9.8%	< 0.001
Maglio et al. [34]	Retrospective	MEPO	*n* = 105	32.7%	24	8.1%	< 0.001
Yılmaz et al. [35]	Retrospective	MEPO	*n* = 31	45.1%	24	3.6%	NS
Chan et al. [23]	Retrospective	MEPO	*n* = 30	46%	44	6%	NS
Bjermer et al. [38]	RCT	RESLI	*n* = 205	N/A	16	0.233 L/s	0.055
Murphy et al. [40]	Open label extension	RESLI	*n* = 1051	1.6 L/s	96	0.4 L/s	N/A
Virchow et al. [41]	Post hoc of 2 RCTs	RESLI	*n* = 723	1.55 L/s	52	0.128 L/s	0.005
Nolasco et al. [42]	Retrospective	BENRA	*n* = 137	38%	24	17%	< 0.001
Pelaia et al. [43]	Retrospective	BENRA	*n* = 22	0.6 L/s	24	0.82 L/s	< 0.001
Castro et al. [47]	RCT	DUPI	*n* = 1902	1.113 L/s	52	0.145 L/s	< 0.001
Wechsler et al. [48]	RCT	DUPI	*n* = 148	N/A	12	0.19 L/s	< 0.05
Pelaia et al. [49]	Prospective	DUPI	*n* = 20	1.47 L/s	4	0.33 L/s	< 0.01
Diver et al. [52]	RCT	TEZE	*n* = 110	1.26 L/s	28	−0.01 L/s	NS
Wechsler et al. [48]	RCT	ITEPE	*n* = 148	N/A	12	0.17 L/s	NS
Wechsler et al. [48]	RCT	ITEPE + DUPI	*n* = 148	N/A	12	0.12 L/s	NS

*BENRA* benralizumab, *DUPI* dupilumab, *ITEPE* itepekimab, *MEPO* mepolizumab, *N/A* not available, *NS* non-significant, *OMA* omalizumab, *RCT* randomised controlled trial, *RESLI* reslizumab, *TEZE* tezepelumab

^a^Absolute values provided as improvements compared to placebo for RCTs

## Conclusions

Prospective RCTs with various biologics are now indicated which are properly powered on small airway outcomes, where patients are selected a priori on the basis of having clinically relevant degrees of SAD. We would duly suggest that such patients might exhibit values for spirometry as FEF_25–75_ < 50%, or oscillometry as X5 < −0.20 kPa/L/s, R5–20 ≥ 0.10 kPa/L/s or AX ≥ 1.0 kPa/L given that such values are associated with poor control and more frequent exacerbations [9, 53]. Ideally, future studies should take into consideration *z*-scores for FEF_25–75_ to account for differences in age and height although in a real-life busy clinic it is perhaps more pragmatic to use absolute cut offs. Oscillometry in particular is easy to perform and effort dependent with validated biological variability values and is therefore eminently suitable for powering such studies in the first instance. In this regard, the ongoing SASAM trial (NCT05040997) is using small airways disease measured by spirometry, body plethysmography, single and multiple breath nitrogen washout and impulse oscillometry as novel endpoints and distinct targets for mepolizumab. The problem for such a trial is deciding on which of the SAD outcomes should be selected as the primary end point in that patients with asthma may for example have relatively well–preserved spirometry with abnormal oscillometry [9, 53]. Another study (NCT03976310) is currently looking at the effects of benralizumab in air trapping, which can be considered a surrogate for small airways disease [10], on high resolution computed tomography imaging as the primary outcome. Tezepelumab is also presently being studied (NCT05280418) to look at its effect in ventilation heterogeneity on hyperpolarised ^129^Xe magnetic resonance imaging as the primary outcome.
